# Medical Society of Tennessee

**Published:** 1841-06

**Authors:** 


MEDICAL SOCIETY OF TENNESSEE.

From the Nashville papers we learn that the late meeting of this Society was well attended, and that its exercises were unusually spirited and interesting. It seems io be gaining with the profession, and is said to be exerting a fine influence upon it. Dr. Hogg, the venerable President, delivered an address which will appear in an early number of our Journal. The annual address was delivered by Dr. L. P. Yandell. A number of instructive cases were reported, and have been placed in the hands of the editors of this Journal for publication. Dr. Brown, of Columbia, was appointed Orator for the next meeting, and an able address may be expected. At the same meeting the Vice President, Dr. Buchanan, also of Columbia, is to deliver an address, according to a standing rule of the Society. In our next number a more detailed account of the proceedings of this spirited, business-doing association may be expected.

